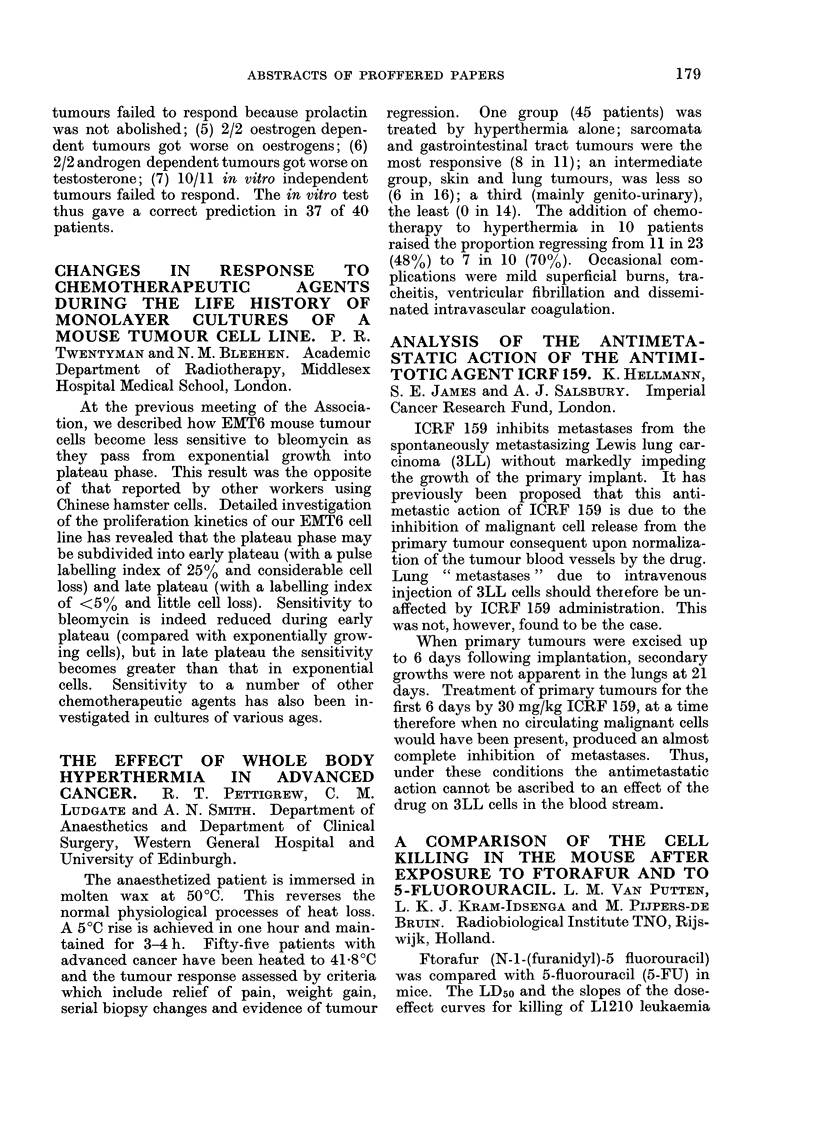# Proceedings: Analysis of the antimetastatic action of the antimitotic agent ICRF 159.

**DOI:** 10.1038/bjc.1974.153

**Published:** 1974-08

**Authors:** K. Hellmann, S. E. James, A. J. Salsbury


### ANALYSIS OF THE ANTIMETASTATIC ACTION OF THE ANTIMITOTIC AGENT ICRF 159.

K. HELLMANN, S. E. JAMES and A. J. SALSBURY. Imperial Cancer Research Fund, London.

ICRF 159 inhibits metastases from the spontaneously metastasizing Lewis lung carcinoma (3LL) without markedly impeding the growth of the primary implant. It has previously been proposed that this antimetastic action of ICRF 159 is due to the inhibition of malignant cell release from the primary tumour consequent upon normalization of the tumour blood vessels by the drug. Lung "metastases" due to intravenous injection of 3LL cells should therefore be unaffected by ICRF 159 administration. This was not, however, found to be the case.

When primary tumours were excised up to 6 days following implantation, secondary growths were not apparent in the lungs at 21 days. Treatment of primary tumours for the first 6 days by 30 mg/kg ICRF 159, at a time therefore when no circulating malignant cells would have been present, produced an almost complete inhibition of metastases. Thus, under these conditions the antimetastatic action cannot be ascribed to an effect of the drug on 3LL cells in the blood stream.